# Advanced Manufacturing Methods for High-Dose Inhalable Powders

**DOI:** 10.3390/pharmaceutics17030359

**Published:** 2025-03-12

**Authors:** Haia A. Al-Assaf, Sofia A. Papadimitriou, Ayesha Rahman, Raj Badhan, Afzal R. Mohammed

**Affiliations:** 1 Aston Pharmacy School, College of Health and Life Sciences, Aston University, Birmingham B4 7ET, UK; 210266519@aston.ac.uk (H.A.A.-A.); r.k.s.badhan@aston.ac.uk (R.B.); 2 National Training Institute, 38446 Vólos, Greece; s.a.papadimitriou@icloud.com; 3 Dentistry, School of Health Sciences, University of Birmingham, Birmingham B5 7EG, UK; a.s.rahman@bham.ac.uk

**Keywords:** dry powder formulation, manufacturing, high-dose powders

## Abstract

Pulmonary drug delivery is governed by three main categories of forces: interparticle forces in the powder formulation, the dispersion forces during inhalation by the device, and deposition forces in the lungs. The interaction between fine inhalable powder particles of the active ingredient is governed by various types of forces, such as capillary forces, electrostatic forces, and van der Waals forces. The different types of inter-particle interactions influence the balance between powder dispersibility and agglomerate stability. The high level of cohesion forces arising from high surface energy of very fine powder hinders powder flowability, leading to issues of agglomeration. Therefore, there is a critical need for advanced manufacturing techniques to overcome the challenges of handling and manufacture of fine cohesive particles, particularly high-dose powders for inhalation. This review will focus on the challenges facing the formulation process of very fine inhalable powder, the various types of existing particle engineering techniques for high-dose powder inhalers, and the characterization techniques employed to analyse the powder characteristics required to meet the acceptance criteria of inhalable preparations.

## 1. Introduction

Inhalation-based therapeutic drug delivery has a history spanning over 4000 years, with its roots tracing back to India and the Middle East [1]. Orally inhaled therapy refers to the type of medications administered to the respiratory system via oral inhalation. Local lung diseases, such as cystic fibrosis, asthma, and chronic obstructive pulmonary diseases (COPDs), can be effectively treated using inhalable preparations that ensure direct delivery of the active pharmaceutical ingredient to the lungs [2]. Administering therapeutic agents for lung diseases as inhalable preparations offers several advantages over systemic therapies. Inhalable drugs can deliver higher concentrations directly to the lungs, which not only enhances their effectiveness but also minimizes systemic side effects by reducing the amount of medication that circulates throughout the body [3]. They help in achieving a faster onset of action and reducing the amount of the administered dose in comparison to the systemic therapy [4]. Also, the pulmonary delivery route avoids any possible inactivation by enzymes and first-pass metabolism that orally administered medication may undergo. Moreover, inhalable formulations can be designed in a way to target systemic diseases and treat them as a non-invasive delivery route [3]. The rapid absorption of active pharmaceutical ingredients (APIs) into the systemic circulation after their direct delivery to lungs is due to the highly vascular epithelial layer of air sacs, with low thickness (0.1–0.2 μm), and large surface areas (~100 m^2^). Furthermore, they achieve rapid absorption into the systemic blood circulation after their deposition within the deep areas of the lungs [2].

In terms of dry powder inhalers (DPIs), there are two approaches to formulation development, including carrier-based and carrier-free formulations. Carrier-based formulations consist of a drug-carrier mixture, in which coarse carrier powder is blended with micronized drug particles. However, carrier-free formulations consist of micronized drug particles only. Formulating carrier-free DPIs comes with several challenges of having only fine drug particles inside the inhaler device due to the tendency of micronized powder to aggregate easily due to their high cohesion forces between particles leading to insufficient dispersion, poor flowability, and low content uniformity [2]. During the formulation process of dry powder for inhalation, stability and dispersibility are the two main features that should be characterized correctly, particularly in cases of biopharmaceutics. Powder dispersibility is affected by the type of excipients in the formulation. Additionally, the ideal choice of excipients enhances powder dispersibility [5]. Generally, powders with a delivered dose ≥ 1 mg are considered high-dose powders. In terms of high-dose inhaled drugs, the solid state of the dry powder defines its aerosol performance and its stability [6]. Drug particles that are customized for pulmonary delivery could achieve central and deep lung deposition when the powder is prepared within an aerodynamic size distribution of 1–5 µm, and more preferably within the 1–3 µm size range. Also, a good stability profile, low retention rates of the formulation with maximal emission from the inhaler, as well as good dispersion are required [7]. During aerosolization, the physicochemical properties of high-dose powder inhalers are influenced by the high concentrations of drug particles loaded in the inhaler device, necessitating advanced manufacturing techniques to enhance the particles’ properties, particularly their dispersibility [1].

The interactions between powder particles of the active ingredient are governed by various types of forces, such as capillary forces, electrostatic forces, and van der Waals forces. The latter mentioned forces are the most common type of forces by number that can be found between dry powder particles [7].

This review will focus on the challenges facing the formulation process of very fine inhalable powder, the various types of already existing particle engineering techniques used to process the formulation powder for high-dose powder inhalers, and the characterization techniques employed to analyse the powder characteristics required to meet the acceptance criteria of inhalable preparations.

## 2. Challenges During Preparation and Formulation of Fine Powder

The deposition of an inhalable formulation within the respiratory tract mainly depends on the shape and size of particles. Mass median aerodynamic diameter (MMAD) refers to the mass of which of 50% of the particle population is smaller than a specific diameter, and the other 50% is greater. Ordinarily, it corresponds to the mean particle diameter of a population. Inhalable particles that are usually with symmetrical and identical particle shape rely on particle size to determine how they deposit within different regions in the lungs. Particles of MMAD values between 0.5 and 5 µm will deposit in the alveoli and small airways, whereas the central airways are expected to trap particles with a size range from 5 to 10 µm. Any particle with an MMAD greater than 10 µm will remain in the oropharyngeal region [8]. It is preferable for drug particles to align within the size range of 1 to 3 μm for better and deeper deposition in the lungs [9].

Pulmonary drug delivery is governed by three main categories of forces: interparticle forces within the powder formulation, dispersion forces during inhalation by the device, and deposition forces in the lungs [1]. The interparticle interactions, such as electrostatic forces, capillary forces, and van der Waals forces, play a crucial role in balancing powder dispersibility and agglomerate stability [10]. Strong interactions between fine particles encourage particle collision, which leads to the generation of larger agglomerates [11]. The interparticle forces can exist between particles of the same material resulting in cohesion forces, or adhesion forces between two different substances such as drug and carrier particles [12]. The maintenance of a good balance between the aforesaid types of forces introduces a significant challenge with regard to developing integrated systems for dry powder inhalers, encompassing both device and pharmaceutical formulation [1].

Alongside other factors such as processing parameters and manufacturing methods, the physico-chemical properties of the drug and the carrier powder control the uniformity and strength of interparticle interactions [13]. Moreover, the physical and chemical properties of the API and the engineered particles determine the extent of van der Waals forces and their dispersion. The physical properties of the final powder comprise surface morphology, including smoothness, rugosity, and purity, as well as particle shape and size distribution. Modification and/or controlling powder physical properties can be achieved via different particle engineering techniques [7]. In terms of surface roughness, it alters the contact area between particles, which in turn leads to stronger van der Waals forces, promoting particle agglomeration. Also, increased surface area as a result of reducing particle size to an inhalable range leads to greater surface interactions when compared to the gravitational force [9].

**Figure 1:**
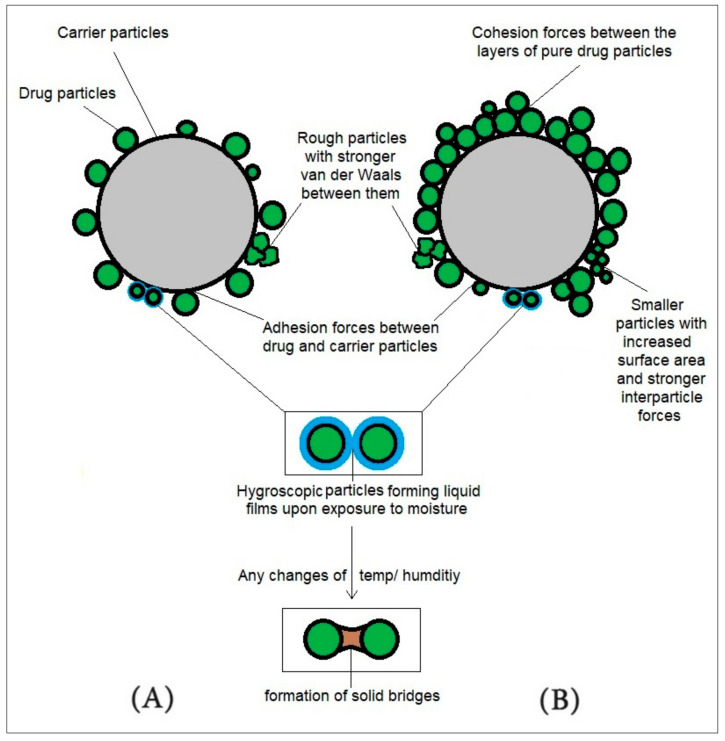
Physico-chemical properties of particles, including roughness, hygroscopicity, and surface area and their influence over the interparticle forces. (**A**) Carrier-based system of low-dose-drug inhaled powder with adhesion forces presenting between the drug-carrier particles. (**B**) Carrier-based system of high-dose-drug inhaled powder with adhesion forces presenting between the drug-carrier particles, as well as cohesion forces between the layers of pure drug particles.

Zeta potential (ZP) is another important parameter that influences the particle properties of very fine powders in pharmaceutical formulations. It indicates the electrical potential at a particle’s interface, which is associated with its surface charge. Furthermore, ZP plays a critical role in determining particle agglomeration, sedimentation, complexation, and interactions with other components in powder formulations [14]. Inadequate control of zeta potential may lead to instability and agglomeration issues [15]. The presence of moisture within a specific hygroscopic formulation contributes to the formation and spreading of liquid films, developing into capillary forces between particles. Moreover, in case of any changes in temperature and/or humidity levels, solid bridges are produced. Thus, issues of chemical instability occur, affecting the dispersion of the powder bed [1]. To illustrate the physico-chemical properties of particles, Figure 1 highlights particle roughness, hygroscopicity, and small particles with large surface areas, and their impact over the interparticle interactions.

The solid state of dry powder, drug, or excipient particles can exhibit either crystalline or amorphous nature. Crystalline particles are packed within long-range structural order, whereas amorphous powders lack the long-range packing order that crystalline powders have. Crystalline particles may possess different forms which are known as polymorphs. Thus, these different solid states express distinct molecular arrangements, as well as different properties. In terms of physico-chemical stability, amorphous structures are known for being unstable and for their high likelihood to crystallize. Transformation of particles from amorphous to crystalline form can affect aerosolization process and contribute to dispersibility issues of powder [6,16].

Similarly, cohesion forces play a vital role in affecting the fluidization behaviour of fine powder. They are influenced by the strength of van der Waals forces and variations in particle size [7]. It is worth mentioning that decreasing particle size increases the degree of cohesiveness between fine particles, leading to their agglomeration [13]. High cohesion forces result in particle agglomeration and increased interparticle mechanical strength. Therefore, they lead to the formation of granular substances. Additionally, strong cohesion forces that outweigh the gravitational forces lead to the formation of strong agglomerates with poor flowability profiles [7]. The agglomerates are more likely to deposit within the oropharynx area, representing the mouth–throat region, leading to unfavourable drug losses [9]. Furthermore, cohesion affects the angle of repose of particles while rotating in preparation drums and affects separation of agglomerates from free powder [17].

Additionally, adhesion forces are considered another major challenge that affect the efficiency of particle de-agglomeration and deposition of dry powder within the respiratory system, particularly the interactive forces between drug and carrier particles [13]. Relevant to mixtures using API and carrier particles, the strength of van der Waals forces depends on the portion of contact area between the type of particles [18]. The impact of adhesion forces over de-agglomeration is studied via the process of converting powder aggregates into fine particle fractions (FPFs). In terms of deep deposition of drug particles in the lungs, the higher values of FPFs reflect better efficiency of the dry powder inhaler device [19]. It is challenging to generate adhesion forces that enable drug particles to be released from the carrier surface during inspiration, while also being strong enough to avoid any segregate formation before the actual use of the inhaler. Correspondingly, poor aerosolization might occur due to strong adhesion forces with partial or incomplete detachment of drug particles from the carrier [10]. In addition, adhesive powder is formed due to van der Waals forces being generated between the formulation powder and the walls of the inhaler device. Attributable to the strong adhesion forces, a high retention time of the dose inside the inhaler is observed [7]. Furthermore, the enlarged aerosol size of ≥10 µm that forms due to adhesive powder formulations in the micron size range, either to each other or to other surfaces, results in poor deposition. The enlarged aerosol will remain in the oropharyngeal region and will not deposit in the lower airways. Achieving effective deposition of therapeutic agents in the deeper areas of the lungs is challenging. Sufficient shear forces are required to break apart particle aggregates by overcoming the adhesion between them. Forceful inhalation by the patient helps to detach these large aggregates and disperse the powder particles. However, this approach may not be suitable for certain age groups, such as children and the elderly. Additionally, patients with COPD are unable to generate enough inspirational forces to overcome any issues of powder aggregation [19]. There are several factors that govern the strength of adhesion forces between particles and adjacent surfaces, including the duration of contact, the structure of the surface and its roughness, and surface contamination. Also, relative humidity and primary contact velocity are encountered [20].

Both cohesion and adhesion forces restrain powder handling, and lead to inefficient powder dispersion, and loss of drug particles in the device [9].

Alongside van der Waals forces, triboelectric forces are another type of force that govern the interactions between particles, and it is challenging to control them [7]. Triboelectric effect results from a generated friction between two materials, followed by their separation [21]. It converts the mechanical energy, which is induced by the mechanically agitated separation, into electrical energy via the arisen potential difference [22]. It may present as occurring between either the same type of materials, or two different types of them [21]. Triboelectric force is not preferable industrially, due to its adverse effects that can lead to electronic damage, ignition, dielectric breakdown, or dust explosions [22]. Relevant to surface characteristics of particles, different surface structures cause static polarized charges generating onto the surfaces. Furthermore, it leads to charge transfer as a suitable strategy to equalize it. Upon separation, the transferred charges attempt to flow back as the electrical difference is terminated. The backflow of charges is not fully accomplished due to the fast separation step that results in a weak electrical conductivity. Both materials keep residues of the polarized charges [18,22]. The surface charge density refers to the amount of exchanged charge between two surfaces per triboelectric generation cycle and it is time and temperature dependent. Faster degradation of surface charge occurs when temperatures are set to be higher. Along with high temperatures, an exponential charge increase within time is observed without counting the initial few minutes [22]. In terms of carrier-based systems, they might contain insulating components that prevent any generated charges from dispersing. Thus, it results in charge accumulation on the surfaces of those particles after each time they are in contact with each other or other surfaces. Indeed, this issue is one of the challenges that is facing the pharmaceutical industry regarding DPI preparation. The complexity of the problem is dependent on the number of involved factors and the number and nature of used materials. It becomes more complex in cases of friction between two insulator materials, or if a metallic material is in contact with an insulator [18].

In carrier-based systems, adhesive forces between microparticles of high-dose powders play a different role compared to microparticles of low-dose powders. This is explained due to variations in particle size distribution amongst drug and carrier particles. As previously mentioned, adhesive powder is formed due to the generation of van der Waals forces between particles and inhaler walls, as well as cohesion forces between pure drug particles. In the case of high-dose powder, drug particles tend to form large lumps with strong cohesion forces in their pure form. The high retention time of the dose in the inhaler is an adverse effect of strong adhesive forces. Additionally, the formation of strong agglomerates with poor flowability is explained by the dominance of cohesion forces over gravitational forces [7].

To demonstrate the differences between low- and high-dose carrier-based systems, Figure 1 shows the types of interparticle forces that influence the powder properties.

**Figure 2:**
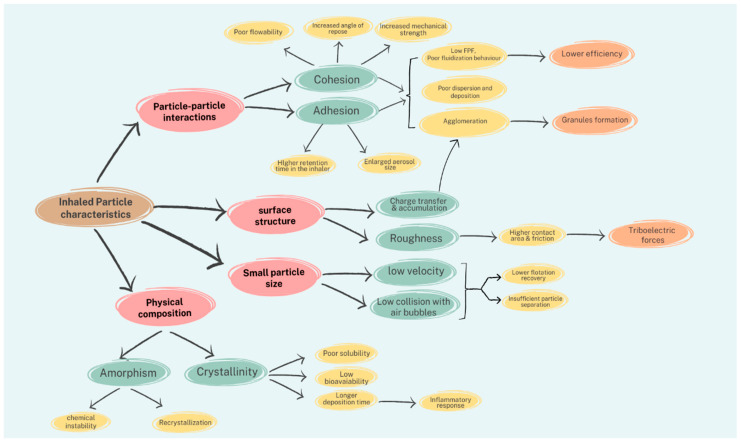
Fine particle characteristics, the challenges associated with fine particle production, and their implications.

To highlight these fundamental challenges of formulating fine particles (Figure 2), interparticle forces, surface structure, composition, and fine particle size are addressed as inevitable characteristics with their implications during the preparation step.

## 3. Particle Engineering Techniques for High-Dose Powders

The high level of cohesion forces arising from high surface energy of very fine powder hinders powder flowability leading to issues of agglomeration. Therefore, balanced interparticle forces using low-energy manufacturing methods are required to develop inhalable formulations of high-dose powder. Particle engineering techniques can be applied to conform ideal fine particle distribution, and good dispersion. Also, they can be used to establish adequate physical and chemical stability during powder preparation and storage [23]. The fundamental physicochemical properties of powder, such as particle shape, roughness and size, and its structure are influenced by the composition and type of manufacturing technique. Also, the aforementioned factors affect the flowability, density, and packing of the resulting particles [24]. This section will review advanced particle engineering methods that have been used to synthesise high-dose inhalation powder formulations.

### 3.1. Mechanofusion

Mechanofusion is considered one of the advanced approaches for preparation of inhalable powders [25]. It changes surface properties of particles by modifying the interparticle forces between drug and excipient particles [26]. It is applied in two forms: either dry or wet coating. In comparison with mechanical wet coating, mechanical dry coating is superior in terms of safety (without the need for solvents), simplicity, and scalability [26,27]. The dry coating process involves preparation of API particles in micrometre size, followed by mixing them with a thin layer of an additive component, such as magnesium stearate, to enhance powder properties, in nanometre size. Magnesium stearate is known for its anti-friction and anti-adherent properties, which enhance the powder flowability by reducing its agglomeration, followed by improved dispersibility profiles [28]. The mechanofusion process is influenced by several operating parameters such as rotational speed, processing time, gap distance, and chamber filling degree, all of which impact coating efficiency and powder flow properties [29]. A higher rotational speed increases energy input, leading to denser particle packing but also raising the risk of excessive adhesion and reduced flowability [30]. Prolonged processing times can enhance coating uniformity but may also cause unwanted temperature rise, leading to particle deformation and stronger adhesion forces [31]. The chamber filling degree, ideally set at 30%, ensures effective mixing without excessive shear stress, while the guest-to-host particle mass ratio determines the coating thickness and uniformity. Torque fluctuations and power consumption during processing provide insights into mixing efficiency, with excessive energy input potentially leading to agglomeration and decreased dispersibility [32]. Generally, mechanofusion apparatus consists of a cylindrical chamber and a process head. Firstly, host and guest particles are installed inside the chamber. At a high speed, both the chamber and the process head rotate with respect to each other. In an effort for the particles to be compressed, they are pushed between the chamber wall and the end of the process head, which creates an impaction with the surface of the process head. Any previously formed agglomerates will continuously be separated when they face the process head during rotation, thus exposing the surfaces of host particles, and allowing the guest particles to cover them [33]. The rotation factor generates thermo-mechanical energy, which aids in the coating step, and the amount of energy is controlled by adjusting the gap size between the process head and the chamber walls [25]. Secondly, using forces of mechanical fusion, the guest particles are smeared over the host particles, and fused into them [34].

Mechanofusion has shown to be a powder-processing technique that provides significant improvement of powder properties, including dispersibility and flowability [25]. In a previous research study, fine particles that had undergone milling were dry coated mechanically using magnesium stearate, producing better profiles of flowability, separation of agglomerates, and particle fluidization [26]. An advantage of using the mechanofusion method is the good control that it offers over the compressive and shear forces [34]. The positive outcome of using mechanofusion is that no remarkable changes are detected over the size of the processed particles [28]. Also, the high shear forces allow the complete coating of an ultra-thin layer of the guest particles over the surface of the host particles [25].

Mechanofusion is a suitable technique to coat larger particles with fine particles producing spherical particles with desirable properties suited for inhalation powders [35]. Compared to other particle engineering techniques, such as milling, mechanofusion proposes good control over the energy input, and advocates coating without any changes to the particle size. Also, the use of mechanical dry coating was successfully employed to suppress the hygroscopicity of moisture-absorbent materials [36].

Research undertaken by Das et al. demonstrated that mechanofusion could be applied to high-dose powder formulations of lactose and 1% of magnesium stearate. Powder dispersion was achieved utilizing two types of inhalers: Monodose Inhaler^®^ (Osnago, Italy) and the Rotahaler^®^ (RH) (Mumbai, India). This occurred at a flow rate of 60 L/min. Also, three dose loads of 10, 25 and 40 mg were tested. After powder processing, a decrease in the work of cohesion, and an increase in the packing fraction, poured, and tapped densities were observed. In addition, the fine particle dose (FPD) exhibited a maximum value of 14 mg for the highest dose load among the three aforementioned doses [37].

In another study initiated by Lakio et al., DPI for high-dose L-arginine (ARG) was optimized using mechanofusion. Magnesium stearate with different percentages and L-leucine were added to formulate several ARG batches. Batches of ARG3 and ARG9 were manufactured with 2%, 20% of magnesium stearate, respectively, whereas 20% of L-leucine was added to batch ARG4. The batches were firstly jet-milled and then mechanofused with the excipients. On the contrary, ARG6 and ARG8 batches underwent jet-milling of API powder with excipients first, followed by mechanofusion. In addition, the ARG10 batch was spray dried followed by mechanofusion. No significant changes were observed in the particle size of spray-dried mechanofused powder, with fewer detected modifications in shape compared to powder processed using ball milling. According to SEM images, ball milling managed to alter particle shape. Furthermore, the ARG10 sample exhibited a significant increase in the powder density. Mechanofused batches showed less cohesion forces in comparison to jet-milled powder. Additionally, all mechanofused batches, including ARG3, ARG6, and ARG8, produced an FPD of >9 mg [36].

### 3.2. Spray Drying

Spray drying is used as a particle engineering technique in the food industry for obtaining dry powder from liquid milk and other liquids. It is a versatile drying technique to solidify liquid preparations such as emulsions, solutions, or suspensions and produce spherical, dry powders. It is an ideal technique that could be used to formulate microparticles for pulmonary delivery [5]. Spray drying has been employed not only for producing drug particles but also for generating carrier materials [38]. The application of spray drying is operated either through closed or open cycles. The closed cycle uses an inert gas, such as nitrogen, to recycle within the drying chamber during the whole spray drying process. On the other hand, the open cycle is considered more cost-effective due to its use of air as the drying gas. This occurs through an open loop that prevents the recirculation of the same air inside the apparatus [39]. The process of spray drying is divided into three stages: it starts with atomization of the liquid preparation, in which the liquid stream goes through nozzles to become droplets; afterwards, the solute or the suspension is separated from the solvent, and dried [40]. The drying step occurs in a hot chamber filled with air or nitrogen to aid with the evaporation of the solvents [6,40]. A rapid precipitation of particles followed by quick evaporation results in dried fine powder [6]. Eventually, the final particles go through a cyclone to be separated and are then transferred to the collection vessels to be collected [40]. Several factors that can be divided into process and formulation variables affect the quality of the spray-dried powders and their properties. These factors include the choice of the method to collect the final product, the time given for drying the atomized droplets, and the rate of drying. Also, the type of the active ingredient, and the type of the atomized nozzles have an influence on the powder properties [38]. Furthermore, the process parameters include inlet temperature, aspirator %, the flow rate of drying gas and liquid feed. Also, liquid feed properties such as surface tension, viscosity, solvent boiling point, density, and concentration affect the resultant particle size and morphology [41]. Moreover, drying parameters and surface activity affect the distribution of elements within the final product [23]. Optimizing spray-drying conditions is crucial, as parameters like drying temperature and feed rate significantly impact product quality, including bulk density, particle size, solubility, and moisture content. Drying temperature directly influences particle morphology, color, and nutrient retention, with higher temperatures leading to lower moisture content but larger particles and reduced bulk density. Feed rate plays a vital role in controlling outlet air temperature, affecting solubility, and drying efficiency. Lower feed rates produce finer particles with reduced residual moisture, while higher feed rates enhance bulk density and productivity [42].

Spray drying is a widely used technique to alter and control with high accuracy the physico-chemical properties of chemical materials. The significance of using the spray drying method to formulate high-dose powders lies in its ability to offer content uniformity of the powder bed, as well as its high level of control over certain particle properties such as density, shape, porosity and surface roughness [43]. Compared to other formulation techniques, such as micronization, spray drying offers good control over the powder density, particle size and morphology [44]. Also, it is an extremely customized technology in controlling the process parameters when compared to other drying techniques, such as freezing drying, tray drying, and rotary drying. The controlled particle and process parameters comprise moisture content, particle size distribution, morphology, chemical structures, and inlet and outlet temperatures [5]. In terms of utilising inert gases in a closed spray-drying cycle, it is useful to employ the spray-drying method when handling oxygen-sensitive materials. Furthermore, the fast-drying step, which might take from seconds to milliseconds, positions spray drying as a suitable method for heat-sensitive materials [45].

Spray drying has been studied in pulmonary delivery to deliver high doses, typically in the range of 10–30 milligrams of the total lung dose [45]. The weak forces between spray-dried particles offer low bulk density and high dispersibility profiles of high-dose powder formulations. High-dose powder of low density disperses better than other particles with high density [46].

Also, the aerosol performance is altered using spray drying, which is due to the impact that the spray drying technique has over the physico-chemical properties of the APIs [44]. The resultant powder has a small mass median diameter and good dispersibility profiles. Thus, no further modifications are required to enhance the dispersibility or decrease the aerodynamic diameter of the spray-dried blend [6].

The spray-drying process can produce corrugated particles, with low density and porosity at the same time. This can happen with or without the addition of other excipients [6]. It is important to highlight that the physical and chemical stability of the final solid particles is better in comparison to their counterpart liquid preparations. The currently available high-dose dry powder inhalers on the market are Aridol^®^/Bronchitol^®^ (Sydney, Australia) and TOBI^®^ Podhaler (Basel, Switzerland) [43].

Practically, the application of the spray-drying technique accompanies several challenges. One of the challenges is its ability to develop dry powder inhaled preparations with good physical stability (particularly controlled amorphous content) and an acceptable aerodynamic performance at the same time [47]. Crystallization is another issue that may occur after spray drying. The level of particle crystallinity relies on two main factors: the type of solvent system used, and the molecules’ nature. When producing inhalable dry powder, amorphous molecules are produced due to the rapid precipitation that follows the quick evaporation step. The production of crystalline molecules is possible but is less likely for drugs with molar mass greater than 300 g mol^−1^. Therefore, significant stability issues have been reported, particularly where drugs are prone to recrystallisation or where supersaturated solutions are spray dried, and collectively, this impacts the physical stability of the resultant spray-dried particles [3,6].

### 3.3. Supercritical Fluid Crystallization

Supercritical fluid crystallization (SFC) is a versatile and efficient technique that can produce fine particles within the micro-size range. It utilizes supercritical fluids (SFs); liquids and gases at temperatures and pressures above their critical points [48]. One of the main key points for using SFC is its association with anti-solvent precipitation. The precipitation step is selective, allowing the formation of pure powders suitable for pulmonary delivery. This is achieved through precipitation, separation, and extraction of unwanted impurities from the final product [49]. SFC is also known as supercritical fluid extraction (SFE) due to the extraction step that removes a soluble component (the extractant) from an insoluble solid or liquid matrix. The use of liquid solvents in SFE is based on the main principle of mass transfer of the required extractant through a solution. The diffusion rate of the solute through the liquid layer of the interface is the controlling factor of the extraction rate [50].

SFs’ own unique features of high compressibility, and their density values are sufficiently capable of providing reasonable solvation power [48]. SFs have lower viscosity, allowing for higher diffusion rates of solutes with better mass transfer when compared to other liquids. Small changes in pressure and temperature offer good control over the solvation power and the density of the SFs. Carbon dioxide is one of the most used gases in pharmaceutical applications due to moderate pressure (73.8 bar) and its low critical temperature of 31.1 °C. Moreover, carbon dioxide is known for being non-toxic, non-flammable, eco-friendly and low cost [48]. One of the major characteristics of carbon dioxide supercritical fluid (SC CO_2_) is that it has good control over extracting and separating organic solvents. Thus, a pure final form of aqueous suspensions, or dry particles is produced. Additionally, a precipitation process at low temperatures, which is easily recycled and clean, is achieved. Reduction in energy, the used amounts of solvents, and the complexity of the manufacturing process are achieved while using SC CO_2_ for particle engineering of sensitive and high-potency particles [49,50].

SFC can enhance powder dispersibility and flowability through its influence over several particle properties, such as shape, density, smoothness, surface porosity, particle size distribution and morphology [1]. Moreover, the use of inert gases with low critical temperatures, such as carbon dioxide, makes SFC suitable for easily oxidised or heat-sensitive materials, including biological ingredients [48,51].

Research conducted by Shekunov et al. compared the aerodynamic forces of salmeterol xinafoate (SX) powders generated by micronization and SFC using CO_2_. The supercritically processed particles demonstrated superior performance; small spherical particles with low bulk density and reduced surface energy were produced. Furthermore, powder deaggregation at relatively low dispersion forces was achieved using SFC leading to the enhanced dispersibility performance of processed powder [52]. Furthermore, a reduction in tensile strength of the aggregates by a factor of seven was observed. SX produced by precipitation in supercritical CO_2_ showed physical properties of low electrostatic charge and low surface energy. Also, decreased adhesion of SX powders to the container walls led to an increased efficiency of drug delivery from dry powder inhalers, which led to a two-fold increase in fine particle fraction in a cascade impactor [52].

One of the key limitations of the precipitation efficiency of SFC is the solubility of the liquid extractant in the supercritical fluid, which can impact the purity and size distribution of the resultant particles [51].

### 3.4. Spray-Freeze Drying

Spray-freeze drying (SFD) is another formulation technique that has been used to produce respirable powders [53]. The process consists of three steps, including droplet atomization, solidification (freezing), and sublimation (drying) at low pressure and temperature. Cold cryogenic liquids such as liquid nitrogen, argon or oxygen are employed to induce solidification of atomized droplets upon contact with them [54,55]. SFD is a technique that combines both principles of spray drying and freeze-drying methods [54]. Initially, a suspension of micro- or nanoparticles is sprayed inside a tank that contains liquid nitrogen. The sprayed droplets are either sprayed on the surface of the liquid nitrogen, or in its bed. Solidification of the sprayed droplets occurs instantly by the time the droplets achieve contact with liquid nitrogen. This can be explained due to the high heat-transfer rate between the sprayed droplets and the liquid nitrogen at a very low temperature of −195.79 °C [53]. Furthermore, the low boiling temperatures of cryogenic liquids encourage the droplets to rapidly freeze [55]. During the spraying step, magnetic stirring is used and set on a mild speed to prevent any agglomerates being formed by the rapid solidification of droplets. After achieving the spraying step, water content, and nitrogen vapour are removed via a vacuum [53]. The process of water removal, or any other solvents, is called lyophilization [53,55].

Spray-freeze-dried particles mostly have excellent dispersion behavior [1]. Porous spherical powder with good aerodynamic performance is produced [53,55].

The good aerodynamic profiles of processed particles have made SFD an attractive method for producing particles for pulmonary delivery [54]. SFD can be used to produce microparticles without any addition of further excipients [24]. It is considered a suitable technique for production of fine particles when it comes to heat-sensitive materials [53]. According to the research carried out by Saluja et al., SFD was used to produce high-dose powder of the stable inulin influenza vaccine for pulmonary immunization. The final spray-freeze-dried formulation showed physical and biochemical stability for at least 3 years at 20 °C [56].

One of the drawbacks of using SFD technique is the potential irreversible damage that might occur to the materials/active pharmaceutical ingredient (particularly biologicals) due to the particle stress at low temperature. Thus, it results in a structural denaturation leading to loss of biological activity and aggregation upon rehydration [55]. Also, powders with high volumes (impact bulk density) are produced, which limits the loading of high drug masses in one dosage form [40]. Another limitation of using SFD is that it is a time-consuming and an expensive method due to the combined use of two major techniques: spray drying, and lyophilization.

### 3.5. Thin-Film Freezing

Thin-film freezing (TFF) is another used technique for the optimization of inhalable high-dose powders [57]. In addition to its use in commercializing small-molecule products, it has been utilized in the food industry, as well as biopharmaceuticals. It is a promising freeze-drying technique for the delivery of pharmaceutical agents for treatment of various pulmonary conditions, such as pneumonia, lung dysfunction, bacterial and fungal infections [58]. Solutions or suspensions of the drug-carrier system can be used as the starting material to generate dry powder particles as the final product [59].

The main principle in TFF is the formation of thin-frozen films of the primary liquid feed on a cryogenically cooled rotating drum, which is subjected to lyophilisation to produce dry powder [58]. Firstly, a feeder solution or suspension that contains both the drug and excipient is prepared [60]. Secondly, in a temperature-controlled chamber with a supercooled solid surface, which is supplied with cryogenic fluid, there is a rotating drum to provide a suitable surface area for the deposition of liquid droplets. Liquid nitrogen is the most commonly used cryogenic fluid in TFF. A microchannel reactor, which works as a processing equipment, transfers the liquid preparation of the required drug and a stabilizer into the processing chamber. Liquid droplets form a wafer or a thin layer by the time they achieve contact with the cryogenically cooled surface and freeze rapidly. Following this, the frozen droplets are lyophilized by transferring the solid pan into a lyophilizer tray to remove solvent particles and produce dry powder [58].

TFF results in modification of surface texture, which produces nanoaggregates with high potency [61]. The processed powder forms a fragile matrix with particles of high surface area, which makes them ideal for use in dry powder inhalers. The matrix of nanoaggregates has low density, which deaggregate into aerosolized particles upon inspiration [57,59].

One of the key advantages of employing TFF is that the freezing step happens at extremely fast rates that prevent any particle growth of the dissolved solute. Additionally, it blocks the generation of any precipitates of amorphous solid dispersion or nanocrystalline aggregates [57]. In comparison to spray-freeze drying and spray drying, TFF engineered particles showed better solubility, stability, morphology, and dissolution profiles, as well as remarkable aerosol performance [58]. The diameter of inhaled microparticles can be >10 microns, with no adverse effects on their delivery to the deep areas of the lungs. Moreover, the advantage of producing microparticles with large geometric diameters greater than 10 microns is their capability to deposit in the deep lung, escape macrophage phagocytosis and extend their retention time [57]. TFF is a preferred method in terms of producing more stable forms of vaccines and biopharmaceuticals, leading to better storage and handling conditions [58].

TFF has many limitations to its use. The type of cosolvent and ratio within the solvent mix can affect the separation of the cryogenic fluid, and the viscosity of the liquid formulation. Furthermore, in a solvent system composed of water and acetonitrile, higher water ratio affects the viscosity of the resultant liquid mix, whereby limited molecular movement and less agglomeration of particles during rapid freezing impacts on particle properties, increasing the probability of agglomeration [62].

In a study using computational models that was conducted by Longest et al., higher efficiency values of drug absorption and content uniformity of the administered dose in the lungs were shown for nanostructured aggregates in comparison to microparticles. Accordingly, the engineered inhalable particles via TFF technique are amorphous particles in a submicron size with high surface areas. Improved bioavailability profiles of drug particles with a history of poor solubility as well as enhanced dissolution rates were encountered [57]. In another study by Moon et al., TFF was used to further optimize inhaled formulation of voriconazole. Mannitol, 3–10% *w*/*w*, was employed as a surface-texture modifying agent to generate crystalline nanoaggregates containing up to 97% *w*/*w* voriconazole. The introduction of low concentrations of mannitol resulted in the reduction in interparticle forces, such as van der Waals forces, which was attributed to changes in surface texture due to layering of fine nanoparticles of mannitol on the surface of voriconazole nanoaggregates. Moreover, the aerodynamic properties of voriconazole nanoaggregates were studied as a lab-scale process (200 mg) and a higher batch process (90 g) and no significant differences were noticed in terms of fine particle fraction, or MMAD between both scales (% of delivered dose, 48.5% vs. 49.5%) and FPF (% of metered dose, 37.0% vs. 35.6%) [61]. According to a recent study by Jara et al., TFF was used for the preparation of an inhaled niclosamide powder for the treatment of COVID-19. The engineered drug particles showed significant targeting of niclosamide to the respiratory system, and particularly the lower respiratory tract. Pharmacokinetic parameters were studied in Syrian golden hamsters and the drug concentrations remained higher for at least 24 h when compared to half maximal inhibitory concentration (IC_50_) and the concentration needed to inhibit viral replication by 90% (IC_90_) [59].

Additionally, TFF had been proven to be an efficient method for the preparation of inhalable powders with no added excipients. High levels of aerosol performance were reported. A recent study was undertaken to study the effects of minimizing excipients in inhalable tacrolimus dry powder formulations. A formulation of 100% *w*/*w* of TFF drug particles showed an FPF (of recovered dose) of 56.34 ± 3.34%, an MMAD of 3.44 ± 0.38 µm, and an emitted fraction (of recovered dose) of 92.94 ± 0.75%. In addition, other research studied the use of TFF remdesivir powder without additional excipients. The FPF (of recovered dose) was 84.25 ± 1.87%, while the emitted fraction (of recovered dose) was 97.10 ± 0.40%. Also, MMAD values were 1.42 ± 0.20 µm, reflecting good aerosol properties of the engineered particles [62].

### 3.6. Isothermal Dry Particle Coating (iDPC)

Isothermal dry particle coating (iDPC) is a blending technology for mixing inhalable powders of high concentrations. Mixtures of carrier-drug particles can be prepared for use in DPIs [63]. Drug particles are directly coated onto the surface of carrier particles by simply mixing both types of particles in a rotating drum. The dry coating process using the iDPC apparatus consists of three stages: de-agglomeration, dispersion, and coating. Agglomerates of fine API and/or coarse particles are distributed as a thin layer along the vessel walls due to the centrifugal speed of the coating process. Additionally, the thin layer of processed powder is fluidized and dispersed with the aid of the nitrogen gas blade. This contributes to further dispersion of the smaller particles throughout the coarse particles. Moreover, dry coating is achieved by the adherence of the fluidised/dispersed particles to the coarse particles [64]. This adherence is based on different types of interparticle forces, such as hydrogen bonding, van der Waals, and electrostatic forces. The process parameters for the iDPC can be easily adjusted including nitrogen flow rate, duration, centrifugal/rotation speed, and batch size. Optimising these parameters can be integrated into the Quality by Design (QbD) framework to control the impact of dry coating on the processed particles. High impaction and mechanical forces are applied during the dry coating process, eliminating the need for using additional solvents or heat [65]. Formulation composition can be adjusted to achieve a partial, monolayer, or multilayer coverage on the surface of carrier particles. In comparison to other conventional shear processes, iDPC does not result in a decrease in aerosolization efficiency, which is commonly experienced with fine powders that are cohesive and tend to agglomerate at high concentrations. A structure of multi-coated layers is formed by first saturating the surface of the carrier particles, followed by cohesive layers of fine API particles coating one another. No alterations in chemical, biological, or physical properties were observed during its use for mixing powders. iDPC application in the pharmaceutical industry resulted in high levels of content uniformity across a wide range of particle types being processed at room temperature [64].

Koner et al. studied the effect of iDPC process parameters on powder blends of fluticasone propionate (FP) formulations with different concentrations ranging from 5 to 90% *w*/*w* of the API and the in vitro aerosol performance was studied using a next-generation impactor. The analytical study was performed using the RS01 inhaler device at a flow rate of 60 L/min, which showed high FPF values between 27.2 and 56.8%. Thus, adjusting iDPC operating conditions can lead to consistent, directed, and predictive aerosol performance of inhalable preparations [64].

The following table (Table 1) summarises the key aspects of the aforementioned particle manufacturing techniques, including their principle, key outcomes, applications, scalability and feasibility and limitations.

## 4. Characterisation Techniques for Inhalation Formulations

### 4.1. Dissolution and Permeability Testing

In vitro dissolution testing is a standard test for the characterization of solid dosage forms to certify the quality of the dosage forms. During the development of orally inhaled dry powder, the focus is to ensure that the API is dispersed from the inhaler device and deposited in the target site within the respiratory tract [66]. Due to the challenges in reproducing in vitro lung conditions, there is currently no official, specific method for testing the release of inhalable powder formulations [67]. The development of a dissolution test suitable for inhalable powder faces several obstacles, including the small amount of aqueous fluids inside the respiratory tract despite its large surface area (>100 m^2^). Also, the clearance mechanisms in the upper respiratory tract differ from those in the lower tract. Phagocytosis by macrophages is the main clearance method of drug particles in the alveoli and lower airways, which are known for having a thin lining of fluid covering them (≥1 µm), whereas mucociliary clearance is a rapid technique for clearing drug particles trapped in a thick mucus film found in the upper respiratory tract [68]. Nevertheless, characterization of the actual differences between pharmaceutical formulations using different excipients can be performed using dissolution [67]. The physicochemical properties of drugs and the physiology of the target site are two factors that determine the dissolution of inhaled drug powder. Modified twin-stage impinger (TSI) was one of the first designed techniques for testing dissolution of inhaled powder. McConville and other co-workers introduced TSI in stage 1 with a 1 mm mesh brass screen. Salbutamol particles were able to diffuse through the mesh into a reservoir filled up with 300 mL of dissolution medium. Closed-loop configuration was used to quantify the drug release of salbutamol [69]. Flow through cell, USP Apparatus 2 (paddle) dissolution apparatus, the DissolvIt system and Franz diffusion cell are other dissolution methods that have been adapted and applied for testing orally inhaled drug particles [66,69].

### 4.2. Particle Size Distribution

The aerodynamic particle size distribution represents the aerosol cloud that consists of drug particles and measures their deposition after inhalation [70]. Particle size distribution can be determined using different techniques. The two most used techniques are light scattering and cascade impactor. The particle size distribution of inhalable particles can be analysed using laser diffraction [71]. In terms of the laser diffraction method, it is a highly precise, reproducible, high throughput and robust technique. Over the last couple of decades, new techniques have been introduced to directly measure the aerodynamic particle size distribution from the aerosol cloud and these include time of flight ‘Aerosizers’, cascade impactors, electrical mobility analysers, spiral centrifuge aerosol spectrometers, sedimentation cells and elutriators and wind sifters [72]. Of these, cascade impactors, the next-generation impactor (NGI) and Anderson cascade impactor (ADI) are widely used methods for in vitro assessment of the drug delivery efficiency of an inhalable preparation [70].

### 4.3. Powder X-Ray Diffraction (PXRD)

Powder X-ray diffraction (PXRD) is one of the characterization methods used to evaluate any changes in the solid state of dry powder upon its exposure to temperature and/or humidity. The principle of PXRD relies on light scattering of X-rays within an order of a long range. Diffraction patterns are produced because of the X-rays being scattered from atoms providing information relating to the arrangement of atoms. Crystalline material generates diffraction patterns that consist of sharp, significant peaks, whereas amorphous materials generally produce a wide halo/peak [73]. PXRD is a useful technique in terms of detecting any deformities in the crystals, orientation parameters, mean crystallite size and micro and macro strain. Furthermore, initial information is provided with regard to texture coefficient and material phases [74].

### 4.4. Scanning Electron Microscopy (SEM)

Scanning electron microscopy (SEM) is considered a powerful surface imaging technique. It is one of the frequently used microscopic methods to determine the size, size distribution and morphological features of powders. SEM is a widely employed technique in testing the quality of the resultant powders for inhalation. Drug distribution, both for low-dose and high-dose drugs, can be visualised using SEM to correlate powder performance (aerosolization) with particle features. Deagglomerated and uniformly distributed drug particles can be distinguished from agglomerates on carrier particles, particularly for low-dose highly cohesive particles. On the other hand, formulations with high doses manufactured using advanced manufacturing methods such as spray drying and TFF can be imaged to assess particle shape, porosity, and possibly surface uniformity [75].

### 4.5. Powder Flowability

Flow properties are relevant to both powder packing and the emptying rate from an inhaler device. Flowability is considered one of the main factors to predict DPI performance both in vitro and in vivo [76]. Obtaining a powder with good flowability can be requested from the powder carrier [70]. A dynamic measurement of powder to determine the angle of repose is used to assess the powder’s flowability. The powder bed is allowed to pass through a funnel from different heights, and the angle at which the particles form after flowing is recorded. The levels of particle deaggregation and aerodynamic dispersion are connected to the varying heights of flowing powder [77]. Both particle size and size distribution affect the flow properties of powder, as well as surface roughness, density, porosity, and particle morphology. The assessment and prediction of flow properties can be achieved by evaluating the modified Hausner ratio. The modified Hausner ratio represents the ratio of poured to compressed bulk powder densities. Moreover, it can be assessed to differentiate between ordered mixtures, which contain up to 5% of micronized lactose [76]. In a study performed to understand the effect of carrier physical properties on DPI performance, carrier with different particles shape were characterised according to their flowability. Commercial lactose (CL), commercial mannitol (CM), cooling crystallised mannitol (CCM), ethanol crystallised mannitol (ECM) and acetone crystallised mannitol (ACM) were used. Poorer flowability was observed for carriers with an elongation ratio. Image analysis was conducted using optical microscopy, and the elongation ratio (ER) was calculated. An ER higher than 5.89 ± 0.2 for ECM compared to 1.62 ± 0.04 for CM indicated more irregularity or elongation in particles of ECM, affecting their flowability properties [78]. In another study, conducted by Kaialy and Nokhodchi (2015), mannitol powder was freeze-dried and was subsequently characterized using different techniques to assess its inhalation performance. It was noticed that better flowability profiles were obtained by larger particles in size with narrower size distribution, lower porosity, and higher bulk and tapped density [79].

### 4.6. Atomic Force Microscopy (AFM)

Atomic force microscopy (AFM) is a technique employed to analyse particles and characterise them on an atomic or nanoscale level [80]. Atomic force microscopy is a good technique to detect piconewton forces that govern specific interactions between particles in solution or dry state [81]. It is utilized to assess different powder characteristics, such as surface rugosity, forces between drug and carrier particles and surface morphology [82]. In a conducted study by Packhaeuser et al., surface morphology of unprocessed and freeze-dried aerosolizable nanocarriers was investigated using AFM. Precise information of particle shape and size was obtained. AFM images showed that the freeze-drying technique has no influence over the morphology and shape of the nanocarrier particles [83]. In another study, budesonide–formoterol inhalation formulation was analysed using AFM, and AFM images revealed that adhesion and cohesion interactions between particles were predominantly governed by van der Waals forces [84]. Moreover, AFM was employed to examine the impact of surface roughness of lactose carrier dry powder on the aerodynamic efficiency. Two different grades of granulated and anhydrous lactose were used, and AFM was used to obtain topographical images of specific lactose size fractions, varying from small anhydrous lactose particles (75–90 μm) to larger particles (250–300 μm). However, measurement of surface roughness of granulated particles was a challenge due to the large changes in surface structure [85].

### 4.7. Solid-State Nuclear Magnetic Resonance (ssNMR) Spectroscopy

Solid-state magnetic nuclear magnetic resonance (ssNMR) is an analytical technique for identification and quantification of very small quantities of amorphous content in crystalline samples. It can detect as low as ~0.5% *w*/*w* of the amorphous content. Also, ssNMR is utilized to examine any molecular interactions, matrix homogeneity, polymorphism, and solid-state transformations. It has been employed for characterization of the solid state of powders by measuring the resonance frequencies of a nuclei NMR can be used to assess single drugs or mixtures, as well as excipients (including polymers) and to evaluate impurity levels, aiding in the interpretation of chemical structure and evaluation of the residual solvents. NMR analysis study has been conducted by Babenkoa et al. to quantify saccharides in dry powder inhaler formulations. Three saccharides, D-(+)-sucrose, D-mannitol, and D-sorbitol., were processed using SFD and SD and NMR was used to explain the differences in pulmonary deposition between SFD D-(+)-sucrose (66.62%) D-mannitol (68.99%) and SD D-(+)-sucrose (57.70%) and SD D-mannitol (49.03%) [86]. Furthermore, solution-state NMR was used to characterise the solid state of co-amorphous ciprofloxacin–quercetin inhalable particles. Firstly, ciprofloxacin–quercetin particles were produced via SD, followed by NMR analysis of intermolecular interactions between both components in solution. A chemical shift in the NMR spectrum of ciprofloxacin suggested an intermolecular interaction between both the drugs and this was attributed the secondary amine group of ciprofloxacin in the region between 9.172 and 8.752 (ppm) [87]. In another study, amorphous and crystalline rifapentine microparticles were prepared and characterised using proton nuclear magnetic resonance (^1^H NMR) where differences between spectra (height of one of the peaks between 1.5 and 1.7 ppm) was attributed to increased water concentration in the crystalline sample [88].

### 4.8. Fourier-Transform Infrared Spectroscopy (FT-IR) and Raman Spectroscopy (FT-Raman) Spectroscopy

FT-IR and FT-Raman spectroscopy are two useful characterization techniques for identification of functional groups in organic compounds. Also, they can be used for quantification of crystallinity, as well as identification and quantification of various polymorphic forms in the DPI formulations. They can distinguish any interactions that occur on a molecular level between excipients and API, such as hydrogen bonding. According to a study by Varun et al., FT-IR was employed to investigate chemical interactions and thermal features of several drug–excipient formulations. Two mixtures of salbutamol sulphate (SS) and aceclofenac (AC) were prepared by mixing each active therapeutic ingredient with lactose monohydrate (LM) in low and high doses (1, 5, 10, 14, 20 and 33 wt.%) in a turbula mixer. Alongside PXRD, and DSC, FT-IR spectra indicated peak shifts after mixing AC-LM, implicating strong surface interactions due to the formation of hydrogen bonds between the drug and excipient [89]. In another study conducted by Kaialy et al., mannitol particles were spray dried and characterized in terms of surface area, size, water content, electrostatic charge, density, and solid state before being blended with albuterol sulphate (AS). FT-IR spectra were recorded for spray-dried mannitol (SDM) and demonstrated similar relative degrees of crystallinity, as well as melting enthalpy when compared to commercial mannitol particles [90]. In another study led by Kaialy et al., recrystallization using non-solvent precipitation was applied to prepare mannitol carrier particles, and ethanol and water were used in the precipitation step. The presence of any molecular changes in recrystallized mannitol during drying or crystallization processes was investigated using FT-IR. D-mannitol was shown to have three polymorphic forms, α, β, and δ. Differences in C–H or O–H stretching vibrations in the regions of 3700–2500 cm^−1^ confirmed the presence of mannitol polymorphs due to structural variations between polymorph β, and δ [91].

### 4.9. Water Content Analysis

Water content analysis is one of the techniques used to analyze moisture content within dry powder inhalation formulations [90]. Alongside other bulk properties, which include particle size distribution, shape, density, relative humidity (RH), temperature, storage time, and formulation composition, water content has an influence over the flowability of materials [92]. Moisture can encourage powder agglomeration and strong adhesion forces, alter the surface of particles, and act as a plasticizing agent [93]. It was noted that particle size influences water uptake, with smaller particles showing higher water uptake compared to larger particles of the same material due to increased surface area [90]. Karl Fischer is one of the methods that can be used to test the water content within inhalable preparations. It can be used to detect the presence of small amounts of water that affect the solid-state properties, powder behaviour of the solid phase, solid-state stability, and capillary condensation [70]. In a previous study conducted by Mitra to study the influence of moisture content on the flow properties of a basundi mix, a popular heat desiccated milk sweet in India, higher moisture content showed dense agglomerated mixtures of basundi powder [92]. Based on findings from a study by Kaialy et al., spray-dried samples of mannitol had undergone water content analysis using the Karl Fischer technique. A negligible quantity of water (0.1 ± 0.1%, *w*/*w*) was detected, indicating the absence of mannitol hydrate form from all the spray-dried samples [90]. In another study led by Wu et al., the impact of environmental humidity was analysed through moisture content analysis of spray-dried lactose samples to investigate recrystallisation in the amorphous state. Samples were stored under different conditions of humidity for 3 or 7 days. It was demonstrated that higher humidity levels (up to 85%) resulted in the emergence of crystal peaks in the DSC patterns, indicating the presence of crystallinity in the samples. In contrast, exposure to 32% RH for 3 days had no effect on the amorphous nature of spray-dried lactose [93].

### 4.10. Zeta Potential Measurement

Zeta potential is a critical parameter in particle characterisation, reflecting the surface charge and stability of particles in suspension. Accurate measurement of zeta potential provides insights into particle interactions, aggregation tendencies, and overall formulation stability. Zeta potential refers to the electrostatic potential at the shear plane, with values around ±30 mV generally indicating well-stabilized particles. Recent advancements have introduced techniques such as Tunable Resistive Pulse Sensing, which offers high-resolution, single-particle analysis of zeta potential, enhancing the robustness and reproducibility of measurements [94]. Tunable Resistive Pulse Sensing (TRPS) allows for the measurement of surface charge on a particle-by-particle basis by analysing the “blockade” signals produced as particles pass through a nanopore, with their velocities being recorded. This technique provides a statistical distribution of zeta potential values within a sample while simultaneously determining both the size and zeta potential of individual nanoparticles [95].

Zeta potential can also be measured by dynamic light scattering (DLS), which uses fluctuations in the scattering of light by particles in suspension to determine their surface charge and size [96]. A study by Kaialy et al. (2013) examined how surface energy and particle size influence the homogeneity of powder formulations. The study used mannitol particles and found that larger mannitol particles exhibited lower surface energy than smaller ones, which could enhance drug content uniformity by reducing energetic barriers to drug-carrier blending [90].

## 5. Conclusions and Perspectives

There are several challenges facing the handling and manufacture of high-dose inhalable powder. Manufacturing methods that include high energy input result in particle agglomeration due to increased surface temperature. Also, physical instability is a common issue while using high shear processes, particularly fragile thermal profiles, where significant high energy leads to a transition between amorphous and crystalline forms. Thus, the use of high temperatures may impact the effectiveness of the therapeutic agents and their stability during storage. Collision and friction forces among particles in high energy techniques can generate heat, causing degradation issues. Consequently, it results in low product yield with potential denaturation of biological materials. The use of high temperatures is one of the critical process parameters that needs to be adjusted correctly to avoid product degradation, crystallization, and agglomeration. Could operating at ambient temperature represent a promising direction for low shear processes?

Also, the high concentrations of high-dose drug powder can exhibit various issues including increased interparticle forces among particles leading to poor flow properties and reducing aerosolization efficiency. Another challenge currently facing the pharmaceutical industry of high-dose DPI formulations is the broad range of product specification. It implies poor control over the manufacturing processes. Good definition of product specification and manufacturing control is crucial for the preparation of consistent batches and safe products. What makes inhalable preparations using the currently available manufacturing techniques yield poor manufacturing control? And which process parameters need to be adjusted to have narrower product specification ranges? The answer pertains to making processes simpler, ensuring stability, and repeatability. This necessitates innovation in advanced particle engineering techniques capable of providing good aerosol performance, with good flowability and dispersibility profiles whilst offering good control over particle properties.

## Figures and Tables

**Table 1:** Overview of advanced manufacturing techniques for preparation and formulation of high dose powder for inhalation.

Manufacturing Technique	Principle of the Method	Key Outcomes	Applications	Scalability and Feasibility	Limitations
Mechanofusion	Host and guest particles rotating inside a cylindrical chamber at high speed, generating thermo-mechanical energy to coat a thin layer of guest particles on the surface of the host particles [25,33].	Improved powder dispersibility, flowability, and separation of agglomerates without altering particle size significantly [26,28]. Achievement of complete, uniform ultra-thin coating [25].	Widely used for preparing inhalable powders and optimizing formulations for high-dose medications such as L-arginine [35,36].	Dry mechanofusion offers scalability and safety, making it suitable for large-scale production of powders without the need for solvents [26,27]. Can be used for moisture-sensitive materials [36]. A feasible method due to its ability to offer fine control over compressive and shear forces, along with minimal change to particle size [28,34].	Alters particle shape and achieves uniformity when scaling up for complex formulations [36].
Spray drying	Liquid preparation is atomized into droplets, rapidly dried with hot gas, and collected as dry fine powder [6,40].	Produces low-density, highly dispersible powder, spherical particles with controlled properties [6,46].	Used for pulmonary drug delivery and carrier material production; applied in high-dose inhalers like Aridol^®^ and TOBI^®^ Podhaler [43,45]. The use of closed spray drying cycle for handling oxygen-sensitive materials [45].	Highly customizable with precise control over powder properties; cost-effective open cycle available (due to its use of air as the drying gas) [5,39,44].	Challenges in maintaining physical stability; risk of recrystallization impacting particle stability [6,47].
Supercritical fluid crystallization (SFC)	Uses supercritical fluids (SFs) to precipitate and extract pure drug particles via anti-solvent precipitation. It is achieved through precipitation, separation, and extraction of unwanted impurities from the final product [48,49].	Produces fine, pure powders with enhanced dispersibility, low surface energy, and improved drug delivery efficiency [1,52].	Suitable for pulmonary drug delivery and particle engineering of heat-sensitive materials [48,51].	Highly efficient with low energy consumption; enables clean and solvent-reduced processing. CO_2_ (one of the most used gases) is non-toxic, cost-effective, and eco-friendly [49,50].	Solubility of liquid extractant in SFs can impact particle purity and size distribution [51].
Spray-freeze drying	Combines spray drying and freeze drying; involves atomization, freezing with cryogenic liquids, and sublimation under a vacuum [53,55].	Produces highly porous, spherical microparticles with excellent dispersibility and good aerodynamic performance [1,55].	Suitable for pulmonary drug delivery, particularly for heat-sensitive materials; used for vaccine formulations with long-term stability [54,56].	Can produce microparticles without excipients; however, high bulk volume limits drug loading; it is time-consuming and expensive due to combined techniques [24,40].	Risk of irreversible damage to biologicals due to particle stress at low temperature, leading to loss of activity; high production costs [55].
Thin-film freezing	The formation of thin frozen film from a liquid preparation on a surface of cryogenically cooled rotating drum, followed by lyophilization and dry powder production [58].	Nanoaggregates with high potency and surface area, a fragile matrix ideal for dry powder inhalers, and low-density particles deaggregate upon inspiration [57,61].	Inhalable pharmaceutical powders, vaccines, and biopharmaceuticals. Also, commercializing small molecule products, and in food industry [58].	Fast freezing prevents particle growth. Better solubility, stability, morphology compared to spray-freeze and spray drying. Production of more stable forms of vaccines, leading to better storage and handling conditions [58].	Solvent mix ratio can affect the separation of the cryogenic fluid and the viscosity of liquid preparation. Could lead to particle agglomeration [62].
Isothermal dry particle coating (iDPC)	Dry coating technique for blending powders. Drug particles adhere to the surface of the carrier particles without the use of solvents or heat [63,65].	Produces high-content uniformity blends, and maintains chemical, biological, and physical stability [64].	Used in inhalable drug formulations, including DPIs, to produce fluticasone propionate (FP) formulations with a high fine particle fraction (FPF) (27.2–56.8%) [64].	Simple process parameters (rotation speed, nitrogen flow, batch size) that can be adjusted for optimal performance. Compatible with QbD framework for process control [65].	A relatively new approach that has not yet been industrialized. Further research is needed to optimize scalability, reproducibility, and regulatory compliance for industrial implementation.
